# Implementing a peer-led psychological self-support program in resource-limited contexts: a pilot in Eastern Cameroon

**DOI:** 10.1192/j.eurpsy.2025.387

**Published:** 2025-08-26

**Authors:** E. Dozio

**Affiliations:** Action contre la Faim, Paris, France

## Abstract

**Introduction:**

In Eastern Cameroon, the psychological needs of both Central African refugees and host communities are significant. Following psychological group interventions over a two-month period, many participants expressed a desire to continue the support process. To meet this need, a self-support protocol was implemented to encourage participants to maintain and strengthen their emotional and social bonds.

**Objectives:**

The aim of this intervention was to enable participants to continue engaging in psychological support autonomously, enhancing their well-being, resilience, and coping mechanisms. The program sought to provide tools for ongoing emotional regulation and peer support, addressing the psychosocial challenges identified during the initial psychological care.

**Methods:**

A flexible, peer-led protocol was developed and implemented after group psychological sessions. Participants were invited to form their own self-support groups, with a recommended size of up to 10 members, meeting weekly for approximately 90 minutes. The structure was intentionally loose to promote autonomy, creativity, and peer leadership. Two initial sessions were facilitated by psychosocial supervisors, while subsequent meetings were primarily observed to assess group evolution. Key dimensions such as emotional well-being, resilience, and social cohesion were measured pre- and post-intervention.

**Results:**

The qualitaive analysis revealed increased cohesion and social connection between group members, especially between refugees and host communities. Participants reported feeling empowered and valued the emotional stability gained through the exercises introduced during the program. However, the evaluation also highlighted challenges, such as maintaining motivation without ongoing supervision and the need for economic opportunities to sustain long-term engagement.

**Conclusions:**

This self-support protocol demonstrated the feasibility and positive impact of peer-led psychological care in contexts with limited resources. The program reinforced emotional resilience, social cohesion, and mutual support, but future iterations should consider integrating economic empowerment initiatives and more structured follow-up to ensure sustained participation.

**Disclosure of Interest:**

None Declared

